# Thematic series on biomedical ontologies in JBMS: challenges and new directions

**DOI:** 10.1186/2041-1480-5-15

**Published:** 2014-03-06

**Authors:** Robert Hoehndorf, Melissa Haendel, Robert Stevens, Dietrich Rebholz-Schuhmann

**Affiliations:** 1 Department of Computer Science, Aberystwyth University, Llandinam Building, SY23 3DB Aberystwyth, UK; 2 OHSU Library and Department of Medical Informatics, Portland, Oregon, USA; 3 Department of Medical Informatics and Epidemiology, Oregon Health & Science University, Portland, Oregon, USA; 4 School of Computer Science, The University of Manchester, Oxford Road, M13 9PL Manchester, UK; 5 Department of Computational Linguistics, University of Zürich, Binzmühlestrasse 14, 8050 Zürich, Switzerland; 6 European Bioinformatics Institute (EMBL-EBI), Wellcome Trust Genome Campus, Hinxton, Cambridge CB10 1SD, UK

## Abstract

Over the past 15 years, the biomedical research community has increased its efforts to produce ontologies encoding biomedical knowledge, and to provide the corresponding infrastructure to maintain them. As ontologies are becoming a central part of biological and biomedical research, a communication channel to publish frequent updates and latest developments on them would be an advantage.

Here, we introduce the JBMS thematic series on Biomedical Ontologies. The aim of the series is to disseminate the latest developments in research on biomedical ontologies and provide a venue for publishing newly developed ontologies, updates to existing ontologies as well as methodological advances, and selected contributions from conferences and workshops. We aim to give this thematic series a central role in the exploration of ongoing research in biomedical ontologies and intend to work closely together with the research community towards this aim. Researchers and working groups are encouraged to provide feedback on novel developments and special topics to be integrated into the existing publication cycles.

## Introduction

This editorial will explore the expectations linked to a growing infrastructure around biomedical ontologies. Since they become an integral part of biological and biomedical research for the annotation of data – its integration, analysis, and visualization [1] – the demand for a place arises in which the scientific community can be made aware of new ontologies, major updates to existing ontologies, development and updates to ontology-based tools, and the discussion of ontology-based methods. The JBMS thematic series on ‘Biomedical Ontologies’, and the annual JBMS Ontology Issue, will fill these gaps and establish a hub of information about biomedical ontologies and their scientific applications.

The role of ontologies in biological and biomedical research has steadily increased in conjunction with the increase in quality and quantity of data that is being collected in all areas of biology. Not only is the number of ontologies increasing, their size growing, their relevance in biomedical research rising and they penetrate more areas of biology and biomedicine; ontologies have also begun to play a key part in the interpretation of the biomedical data as well as inspire the development of new tools for end users and new analysis methods for biomedical scientists. As a result, data integration and interoperability has become a relevant cost factor in the execution of big data projects and has been acknowledged by national and international projects, for example by the Elixir initiative, which aims to establish a biomedical IT infrastructure across Europe [2]. The development and application of ontologies will be an integral part of such an infrastructure for the main reason that data interoperability requires tools to explicitly describe the semantics of terms used to characterize the features of data, and ontologies are widely used to fill this role.

## Which ‘ontology’ did you mean?

There has been considerable debate in the ontology research community as to what constitutes an ontology in biology [3-5] and what properties an ontology should have. Traditional axes of classification for ontologies include the expressivity of the language used to develop and distribute the ontologies, the applications for which the ontologies are intended (i.e. who uses the ontology and how) and the domain covered by the ontology. Arguments pertain 

(a) To the *degree of formality* of the language used to express the information in an ontology, i.e., whether a formal language such as the Web Ontology Language (OWL) [6] is used or a graph-based representation without explicit formal semantics,

(b) To the *complexity* of the ontology description, i.e., whether rich axioms and relations are used or whether a taxonomy, accompanied with textual definitions of classes in an ontology, is sufficient,

(c) To the *interpretation* of what constitutes a “class” or “relation” in an ontology, i.e., whether a class in an ontology refers to something in the world or to a mental construct, and

(d) To the *orthogonality* of the content, i.e., what content has been incorporated from other ontologies and for which purposes.

Depending on the intended applications, artifacts called “ontologies” are developed with any combination of these properties.

In the JBMS thematic series on Biomedical Ontologies, we employ a broad interpretation of “ontology” and include artifacts that primarily provide vocabularies for the purpose of data annotation as well as formal theories that provide a rich representation of certain aspects of biomedicine. To annotate data within a database, a taxonomy of classes with labels and textual definitions is often sufficient, while more expressive formal constructs would be required if the ontology is developed to verify data integrity.

### Representing ontologies

The annotation of research data using an ontology enables integration of data both within a database and across multiple databases [1]. Ontologies provide a controlled set of classes together with an explicit (formal or informal) representation of their meaning, a hierarchy between these classes and complex axiom patterns (“relations”) [7] between the classes, and ontologies facilitate data integration when shared across multiple databases. The taxonomic relations allow integration through general or specific aspects even if exact matches between data items can not be identified; and axioms between classes serve as complex relations that facilitate further data integration.

Today, most biomedical ontologies are developed in shared formal languages, either the OBO Flatfile Format [8] or the Web Ontology Language (OWL) [6]. Both languages are tightly coupled and thus allow translations between them [9,10] so that the OBO Flatfile Format can now be considered to be a fragment of OWL [8].

The expressivity of a biomedical ontology is determined by the particular subset of OWL that is being used to formulate the ontologies, and serves as a major distinguishing factor. It characterizes the knowledge that can be expressed (such as whether the ontology may contain contradictions) and determines the complexity of general tasks such as querying the ontology and categorizing data with the ontology. OWL 2 comprises three emerging profiles (OWL EL, OWL QL and OWL RL) apart from OWL DL [11]. The OWL EL profile forms a subset which (a) allows to specify a taxonomy between classes (i.e., to state that one class is the subclass of another), (b) existential restrictions (i.e., to state that instances of one class must stand in a relation to some instance of another class), and (c) disjointness of classes (i.e., to state that two classes cannot share any instances), and has been found useful for a significant number of biomedical ontologies [12-15].

### Domains of ontologies and their applications

Ontologies are of particular importance in domains in which large volumes of data are being generated, and the emergence of high-throughput technologies has increased the importance of ontologies in some domains. In the 1990s, research on discovering gene functions in diverse organisms required a means to standardize gene functions for comparison within and across multiple organisms: this need induced the development of the Gene Ontology (GO), which turned into one of the most important resources in genomics research [16]. In a similar way, the Sequence Ontology (SO) [17] emerged as a response to the availability of more and more sequencing data, and to provide compatibility between different data formats for biological sequences and their features.

Different anatomy ontologies specify the organismal components for multiple species, and – on a smaller scale of granularity – the developmental relations and features of cell types are characterized by the Celltype Ontology [18]. Phenotype ontologies are also available for multiple species and are widely used for the annotation of the abnormalities observed in mutagenesis experiments [19-21] as well as for the characterization of diseases and drug effects [22].

Further domains covered comprise chemical entities to annotate drugs and theirs biological activities [23], structures, and pharmaceutical applications [23,24] for data interoperability [25], and ontologies for experimental settings, e.g., the BioAssay Ontology [26], the Experimental Factor Ontology [27], the eagle-i ontology [28] and the Ontology of Biomedical Investigations [29], capture the biomedical metadata to characterize experiments. Similarly, ontologies for environmental conditions denote data samples and their surroundings upon their encounter [27,30]. Ontologies are also being used to annotate and classify journal articles [31,32], pathways [33], and specific biological entities [34].

Ontologies, together with their annotations, are extensively used in the analysis of biomedical data, for example in the form of Gene Set Enrichment Analysis (GSEA) [35] for the interpretation of gene expression datasets. GSEA makes use of the structure of the Gene Ontology to identify statistically over- or under-represented classes based on gene expression observed in two biological states. Similar methods are also applied to other ontologies such as the Human Disease Ontology [36], the Neuro Behavior Ontology [37], or even the full set of ontologies contained in BioPortal [38].

Another analysis method relying on ontologies is to compare data items and identify meaningful biological relations between them based on semantic similarity [39]. This approach has been applied to identify protein-protein interactions [40], classify chemicals [41], suggest candidate genes involved in diseases [42,43] and repurpose drugs [44,45]. When applying semantic similarity to compare two data items, the choice of ontology determines the kind of similarity that is revealed: using GO will provide *functional* similarity, chemical entities from ChEBI will provide *chemical structure* similarity, and using phenotype ontologies will result in *phenotypic* similarity.

The integration of multiple ontologies – in particular from different domains – can reveal relations between annotated data items. For example, anatomy ontologies for cross-species comparisons – linking homologous or analogous anatomical structures – can be used to transfer and compare annotations for multiple species [15,46]. For this purpose, the UBERON anatomy ontology [15] was developed. It enables cross-species phenotype representations that have been applied to deciphering human GWAS data based on comparisons with mouse model phenotypes [47] as well as the prioritization of candidate genes and drug targets based on data from model organisms [42,44,48,49].

Additionally, the rich axiom systems of some ontologies help to verify and classify data according to constraints on biological entities expressed in the ontologies. One example of such an application has been the classification of proteins using ontologies [50], in which an ontology provides rules according to which decisions about the protein family are made. The same, or similar, constraints expressed in ontologies can be used to verify data, i.e., determine whether a data item complies with the constraints expressed in the ontology or not [51].

## The main challenges for research in biomedical ontologies

### Evaluation of ontologies and the development of a robust research methodology

Establishing effective methods to evaluate ontologies – both qualitatively and quantitatively, if possible – towards fitness for a purpose is a major challenge in ontology research [52]. Determining the “best” ontology for a given purpose becomes important, and criteria such as the ontology structure, formality, its complexity, its coverage, as well as the amount of data annotated with it contribute to this decision. Effective methods for evaluation are particularly required for domains in which multiple ontologies overlap in their content and intended applications, such as for human diseases where ICD, MeSH, SNOMED CT, the Human Disease Ontology [53], the Human Phenotype Ontology [22], the Unified Medical Language System (UMLS) [54], and more specific ontologies such as the Infectious Disease Ontology [55], Malaria ontology [56], etc. are being used.

The research methodology underlying the development of biomedical ontologies will also improve when effective evaluation criteria are being applied. The Ontology Summit [57] has addressed this need with the topic “Ontology Evaluation Across the Ontology Lifecycle” in 2013, and ontology evaluation featured prominently in panel discussions at the International Conference on Biomedical Ontologies 2013 (ICBO) and will play a prominent role at ICBO2014. The JBMS thematic series on Biomedical Ontologies will follow the community discussions to address ontology evaluation principles and methods, and their instantiation in community-agreed guidelines and standards.

### Standards and Interoperability: Linked Data and beyond

Efficient reuse of ontologies, and the knowledge they contain, in the organization of open, linked data possibly accessible through multiple public interfaces (SPARQL endpoints) from different data providers is another challenge [58]. The main task is to balance the complexity of processing and querying ontologies, which commonly require the use of an automated reasoner, with the need to efficiently query large, linked datasets. In particular when multiple ontologies are used to annotate datasets and automated reasoning over these ontologies provides the means for finding relations between the classes in these ontologies, the need for an infrastructure to support combined queries over ontologies with queries over linked data using SPARQL arises.

Recently, some applications have come forward in which automated reasoning is used to answer complex queries over ontologies and subsequently retrieve data [59-61]. At the same time, major providers of biological and biomedical data such as the European Bioinformatics Institute (https://www.ebi.ac.uk/rdf/) and UniProt (http://beta.sparql.uniprot.org/) provide access to their content through public SPARQL endpoints. In the future, we expect exciting applications that combine reasoning over ontologies in ontology repositories, such as the Ontology Lookup Service [62], BioPortal [63] or OntoBee [64], with (federated) SPARQL queries and provide a genuinely knowledge-driven way for exploring linked biomedical data.

### Knowledge-based analysis of biomedical data

Integration of ontologies – and the knowledge they contain – in the analysis of biological and biomedical data is yet another challenge. Ontologies have been successfully integrated with biomedical analysis pipelines [35,39,65,66]. However, these analysis methods mainly exploit the ontologies’ taxonomy and often make use of the axioms and constraints only implicitly.

Many ontologies contain a lot more information than taxonomic relationships, and some recent work has begun to exploit some additional information – disjointness between classes in an ontology – to improve computation of semantic similarity [67]. How the rich information that is further contained in formalized ontologies can be incorporated in the analysis of biomedical data remains a research question, and novel methods will likely appear as the infrastructure and tool support around ontologies evolves.

## The JBMS thematic series on “Biomedical ontologies”

The JBMS thematic series on Biomedical Ontologies will provide the venue for publishing research about biomedical ontologies, their development, integration and quality assurance. On a regular basis, we will have open calls for papers on specific topics, and we welcome community input for important challenges to address.

The annual JBMS Ontology Issue will become a central part of the thematic series where we focus on ontologies that have already been demonstrated to be useful for scientific applications. The Ontology Issue is intended for a wide audience of readers; it does not specifically target researchers in ontology, but rather biological and biomedical researchers who may want to apply ontologies in their domain and require an overview over the currently available artifacts they can already use. In the Ontology Issue, new ontologies can be described as well as updates to existing ontologies. Updates in regular intervals produce a better understanding of the progress in developing an ontology and the major changes to its content, structure and applications.

In the future, we aim to establish another regular call in which ontology-based tools and applications will be described and updates to these tools published. Additionally, the thematic series will provide a venue to publish conference and workshop papers, and interested working groups are encouraged to suggest special topics or to contribute to existing publication cycles.

We aim to make the JBMS thematic series on Biomedical Ontologies take a central role in the exploration of current research in biomedical ontologies, and we intend to work closely with the research community to achieve this aim. All researchers are invited to express ideas and demands, ask for feedback on topics, and provide suggestions for novel developments.

## Competing interests

The authors declare that they have no competing interests.

## Authors’ contributions

RH and DRS have drafted the first manuscript version. MH and RS have contributed valuable improvements to the manuscript. All authors read and approved the final manuscript.

## References

[B1] Rebholz-Schuhmann D Oellrich A Hoehndorf R Text-mining solutions for biomedical research: enabling integrative biology Nat Rev Genet 2012 13 12 829 839 10.1038/nrg3337 23150036

[B2] Crosswell LC Thornton JM Elixir: a distributed infrastructure for European biological data Trends Biotechnol 2012 30 5 241 242 10.1016/j.tibtech.2012.02.002 22417641

[B3] Merrill GH Ontological realism: methodology or misdirection? Appl Ontol 2010 5 2 79 108 doi:10.3233/AO-2010-0076 10.3233/AO-2010-0079 PMC3104413 21637730

[B4] Smith B Ceusters W Ontological realism: a methodology for coordinated evolution of scientific ontologies Appl Ontol 2010 5 139 188 21637730 10.3233/AO-2010-0079 PMC3104413

[B5] Lord P Stevens R Adding a little reality to building ontologies for biology PLoS ONE 2010 5 9 12258 doi:10.1371/journal.pone.0X00000.012258 10.1371/journal.pone.0012258 PMC2933225 20838431

[B6] Grau B Horrocks I Motik B Parsia B Patelschneider P Sattler U OWL 2: The next step for OWL Web Seman Sci Serv Agents World Wide Web 2008 6 4 309 322 doi:10.1016/j.websem.2008.05.001 10.1016/j.websem.2008.05.001

[B7] Smith B Ceusters W Klagges B Köhler J Kumar A Lomax J Mungall C Neuhaus F Rector AL Rosse C Relations in biomedical ontologies Genome Biol 2005 6 5 46 doi:10.1186/gb-2005-6-5-r46 10.1186/gb-2005-6-5-r46 PMC1175958 15892874

[B8] Horrocks I OBO flat file format syntax and semantics and mapping to OWL web ontology language Technical report University of Manchester (March 2007). http://www.cs.man.ac.uk/~horrocks/obo/

[B9] Moreira DA Musen MA Obo to owl: a protege owl tab to read/save obo ontologies Bioinformatics 2007 23 14 1868 1870 doi:10.1093/bioinformatics/btm258 10.1093/bioinformatics/btm258 17496317

[B10] Golbreich C Horrocks I Parsia B, Kalyanpur A, Golbreich C, Golbreich C , Kalyanpur A , Parsia B The obo to owl mapping, go to owl 1.1! In, Proceedings of, OWL 1.1! Experiences and Directions 2007 (OWLED-2007) 2007 CEUR-WS.org

[B11] Motik B Grau B. C Horrocks I Wu Z Fokoue A Lutz C Owl 2 web ontology language: profiles W3C Recomm 2009 27 61

[B12] Dentler K Cornet R Teije A Keizer N Comparison of reasoners for large ontologies in the owl 2 el profile Semant Web 2011 2 2 71 87 doi:10.3233/SW-2011-0034

[B13] Horridge M Parsia B Sattler U The State of Bio-Medical Ontologies 2011 http://bio-ontologies.knowledgeblog.org/135

[B14] Hoehndorf R Dumontier M Oellrich A Wimalaratne S RebholzSchuhmann D Schofield P Gkoutos GV A common layer of interoperability for biomedical ontologies based on OWL EL Bioinformatics 2011 27 7 1001 1008 10.1093/bioinformatics/btr058 21343142 PMC3065691

[B15] Mungall C Torniai C Gkoutos G Lewis S Haendel M Uberon, an integrative multi-species anatomy ontology Genome Biol 2012 13 1 5 doi:10.1186/gb-2012-13-1-r5 10.1186/gb-2012-13-1-r5 PMC3334586 22293552

[B16] Ashburner M Ball CA Blake JA Botstein D Butler H Cherry MJ Davis AP Dolinski K Dwight SS Eppig JT Harris MA Hill DP Tarver LI Kasarskis A Lewis S Matese JC Richardson JE Ringwald M Rubin GM Sherlock G Gene ontology: tool for the unification of biology Nat Genet 2000 25 1 25 29 doi:10.1038/75556 10.1038/75556 10802651 PMC3037419

[B17] Mungall CJ Batchelor C Eilbeck K Evolution of the sequence ontology terms and relationships J Biomed Inform 2011 44 1 87 93 10.1016/j.jbi.2010.03.002 20226267 PMC3052763

[B18] Bard J Rhee SY Ashburner M An ontology for cell types Genome Biol 2005 6 2 doi:10.1186/gb-2005-6-2-r21 10.1186/gb-2005-6-2-r21 PMC551541 15693950

[B19] Mabee PM Ashburner M Cronk Q Gkoutos GV Haendel M Segerdell E Mungall C Westerfield M Phenotype ontologies: the bridge between genomics and evolution Trends Ecol Evol (Personal edition) 2007 22 7 345 350 doi:10.1016/j.tree.2007.03.013 10.1016/j.tree.2007.03.013 17416439

[B20] Gkoutos GV Green EC Mallon AM Hancock JM Davidson D Building mouse phenotype ontologies Pac Symp Biocomput 2004 178 189 14992502 10.1142/9789812704856_0018

[B21] Smith CL Eppig JT The mammalian phenotype ontology as a unifying standard for experimental and high-throughput phenotyping data Mammal Genome 2012 23 9–10 653 668 10.1007/s00335-012-9421-3 PMC3463787 22961259

[B22] Köhler S Doelken SC Mungall CJ Bauer S Firth HV Bailleul-Forestier I Black GC Brown DL Brudno M Campbell J FitzPatrick DR Eppig JT Jackson AP Freson K Girdea M Helbig I Hurst JA Jähn J Jackson LG Kelly AM Ledbetter DH Mansour S Martin CL Moss C Mumford A Ouwehand WH Park SM Riggs ER Scott RH Sisodiya S The human phenotype ontology project: linking molecular biology and disease through phenotype data Nucleic Acids Res 2014 42 D1 966 974 10.1093/nar/gkt1026 PMC3965098 24217912

[B23] Degtyarenko K Matos P Ennis M Hastings J Zbinden M McNaught A Alcantara R Darsow M Guedj M Ashburner M ChEBI: a database and ontology for chemical entities of biological interest Nucleic Acids Res 2008 36 suppl 1 D344 D350 17932057 10.1093/nar/gkm791 PMC2238832

[B24] Hettne KM Williams AJ van Mulligen E M Kleinjans J Tkachenko V Kors JA Automatic vs. manual curation of a multi-source chemical dictionary: the impact on text mining J Cheminform 2010 2 4 10.1186/1758-2946-2-4 20525267 PMC2890529

[B25] Gaulton A Bellis LJ Bento AP Chambers J Davies M Hersey A Light Y McGlinchey S Michalovich D Al-Lazikani B Overington JPC Chembl: a large-scale bioactivity database for drug discovery Nucleic Acids Res 2012 40 D1 1100 1107 doi:10.1093/nar/gkr777. http://nar.oxfordjournals.org/content/40/D1/D1100.full.pdf+html 10.1093/nar/gkr777 PMC3245175 21948594

[B26] Visser U Abeyruwan S Vempati U Smith RP Lemmon V Schürer SC Bioassay ontology (bao): a semantic description of bioassays and high-throughput screening results BMC Bioinform 2011 12 257 doi:10.1186/1471-2105-12-257 10.1186/1471-2105-12-257 PMC3149580 21702939

[B27] Malone J Holloway E Adamusiak T Kapushesky M Zheng J Kolesnikov N Zhukova A Brazma A Parkinson H Modeling sample variables with an Experimental Factor Ontology Bioinformatics 2010 26 1112 1118 10.1093/bioinformatics/btq099 20200009 PMC2853691

[B28] Haendel M Wilson M Torniai C Segerdell E Shaffer C Frost R Bourges D Brownstein J McInnerney K Eagle-i: Making invisible resources, visible J Biomol Tech: JBT 2010 21 3 Suppl 64

[B29] Brinkman RR Courtot M Derom D Fostel JM He Y Lord P Malone J Parkinson H Peters B Rocca-Serra P Ruttenberg A Sansone S-A Soldatova LN Stoeckert CJJr Turner JA Zheng J OBIc: Modeling biomedical experimental processes with obi J Biomed Seman 2010 1 Suppl 7 10.1186/2041-1480-1-S1-S7 PMC2903726 20626927

[B30] Buttigieg PL Morrison N Smith B Mungall C Lewis S The environment ontology: contextualising biological and biomedical entities J Biomed Seman 2013 4 1 43 10.1186/2041-1480-4-43 10.1186/2041-1480-4-43 PMC3904460 24330602

[B31] Doms A Schroeder M GoPubMed: exploring PubMed with the Gene Ontology Nucleic Acids Res 2005 33 Web Server issue 783 786 10.1093/nar/gki470 PMC1160231 15980585

[B32] Gaudan S Yepes AJ Lee V Rebholz-Schuhmann D Combining evidence, specificity, and proximity towards the normalization of gene ontology terms in text EURASIP J Bioinform Syst Biol 2008 3427 3446 10.1155/2008/342746 PMC3171395 18437221

[B33] Joshi-Tope G Gillespie M Vastrik I D’Eustachio P Schmidt E de Bono B Jassal B Gopinath GR Wu GR Matthews L Lewis S Birney E Stein L Reactome: a knowledgebase of biological pathways Nucleic Acids Res 2005 33 Database issue 428 432 doi:10.1093/nar/gki072 10.1093/nar/gki072 PMC540026 15608231

[B34] Chelliah V Laibe C Novère NL Biomodels database: a repository of mathematical models of biological processes Methods Mol Biol 2013 1021 189 199 10.1007/978-1-62703-450-0_10 23715986

[B35] Subramanian A Tamayo P Mootha VK Mukherjee S Ebert BL Gillette MA Paulovich A Pomeroy SL Golub TR Lander ES Mesirov JP Gene set enrichment analysis: A knowledge-based approach for interpreting genome-wide expression profiles Proc Nat Acad Sci USA 2005 102 43 15545 15550 doi:10.1073/pnas.0506.580102. http://www.pnas.org/content/102/43/15545.full.pdf+html 10.1073/pnas.0506580102 16199517 PMC1239896

[B36] LePendu P Musen M Shah N Enabling enrichment analysis with the human disease ontology J Biomed Inform 2011 S31 S38 21550421 10.1016/j.jbi.2011.04.007 PMC3392036

[B37] Hoehndorf R Hancock JM Hardy NW Mallon A-M Schofield PN Gkoutos GV Analyzing gene expression data in mice with the neuro behavior ontology Mammal Genome 2013 1 9 10.1007/s00335-013-9481-z 24177753

[B38] Wittkop T TerAvest E Evani U Fleisch K Berman A Powell C Shah N Mooney S STOP using just GO: a multi-ontology hypothesis generation tool for high throughput experimentation BMC Bioinform 2013 14 1 53 10.1186/1471-2105-14-53 PMC3635999 23409969

[B39] Pesquita C Faria D Falcao AO Lord P Couto FM Semantic similarity in biomedical ontologies PLoS Comput Biol 2009 5 7 1000443 doi:10.1371/journal.pcbi.1000443 10.1371/journal.pcbi.1000443 PMC2712090 19649320

[B40] Guzzi PH Mina M Guerra C Cannataro M Semantic similarity analysis of protein data: assessment with biological features and issues Brief Bioinform 2011 13 5 569 585 doi:10.1093/bib/bbr066. http://bib.oxfordjournals.org/content/early/2011/12/02/bib.bbr066.full.pdf+html 22138322 10.1093/bib/bbr066

[B41] Ferreira JD Couto FM Semantic similarity for automatic classification of chemical compounds PLoS Comput Biol 2010 6 9 1000937 doi:10.1371/journal.pcbi.1000937 10.1371/journal.pcbi.1000937 PMC2944781 20885779

[B42] Smedley D Oellrich A Köhler S Ruef B Mungall C Project SMG Phenodigm: analyzing curated annotations to associate animal models with human diseases Database 2013 doi:10.1093/database/bat025. http://database.oxfordjournals.org/content/2013/bat025.full.pdf+html 10.1093/database/bat025 PMC3649640 23660285

[B43] Hoehndorf R Schofield PN Gkoutos GV Phenomenet: a whole-phenome approach to disease gene discovery Nucleic Acids Res 2011 39 18 119 10.1093/nar/gkr538 21737429 PMC3185433

[B44] Hoehndorf R Hiebert T Hardy NW Schofield PN Gkoutos GV Dumontier M Mouse model phenotypes provide information about human drug targets Bioinformatics 2013 http://bioinformatics.oxfordjournals.org/content/early/2013/10/23/bioinformatics.btt613.full.pdf+html 10.1093/bioinformatics/btt613 PMC3933875 24158600

[B45] Gottlieb A Stein GY Ruppin E Sharan R PREDICT: a method for inferring novel drug indications with application to personalized medicine Molecular Syst Biol 2011 7 496 doi:10.1038/msb.2011.26 10.1038/msb.2011.26 PMC3159979 21654673

[B46] Niknejad A Comte A Parmentier G Roux J Bastian FB Robinson-Rechavi M vhog, a multispecies vertebrate ontology of homologous organs groups Bioinformatics 2012 28 7 1017 1020 10.1093/bioinformatics/bts048 22285560 PMC3315709

[B47] Robinson P Köhler S Oellrich A Project SMG Wang K Mungall C Lewis SE Washington N Bauer S Seelow DS Krawitz P Gilissen C Haendel M Smedley D Improved exome prioritization of disease genes through cross species phenotype comparison Genome Res 2013 doi:10.1101/gr.160325113. http://genome.cshlp.org/content/early/2013/10/25/gr.160325113.full.pdf+html 10.1101/gr.160325.113 PMC3912424 24162188

[B48] Washington NL Haendel MA Mungall CJ Ashburner M Westerfield M Lewis SE Linking human diseases to animal models using ontology-based phenotype annotation PLoS Biol 2009 7 11 1000247 10.1371/journal.pbio.1000247 PMC2774506 19956802

[B49] Robinson PN Köhler S Oellrich A Smedley D Sanger Mouse Genetics Project Improved exome prioritization of disease genes through cross-species phenotype comparison Genome Res 2014 24 2 340 8 10.1101/gr.160325.113 24162188 PMC3912424

[B50] Wolstencroft K Lord P Tabernero L Brass A Stevens R Protein classification using ontology classification Bioinformatics 2006 22 14 530 538 10.1093/bioinformatics/btl208 16873517

[B51] Hoehndorf R Dumontier M Gennari JH Wimalaratne S de Bono B Integrating systems biology models and biomedical ontologies BMC Syst Biol 2011 5 1 124 10.1186/1752-0509-5-124 21835028 PMC3170340

[B52] Hoehndorf R Dumontier M Gkoutos GV Evaluation of research in biomedical ontologies Brief Bioinform 2012 14 6 696 712 22962340 10.1093/bib/bbs053 PMC3888109

[B53] Schriml LM Arze C Nadendla S Chang YWW Mazaitis M Felix V Feng G Kibbe WA Disease Ontology: a backbone for disease semantic integration Nucleic Acids Res 2011 40 D1 D940 D946 22080554 10.1093/nar/gkr972 PMC3245088

[B54] Bodenreider O The Unified Medical Language System (UMLS): integrating biomedical terminology Nucleic Acids Res 2004 32 Database issue 267 270 10.1093/nar/gkh061 PMC308795 14681409

[B55] Goldfain A Smith B Cowell L Dispositions and the infectious disease ontology Proceedings of Formal Ontologies in Information Systems (FOIS) 2010 IOS Press 400 413 ISBN 978-1-60750-534-1

[B56] Topalis P Dialynas E Mitraka E Deliyanni E Siden-Kiamos I Louis C A set of ontologies to drive tools for the control of vector-borne diseases 2010 10.1016/j.jbi.2010.03.012 PMC2953594 20363364

[B57] Gruninger M Obrst L Big data and semantic web meet applied ontology Symposium at the National Institute of Standards (NIST) Gaithersburg, Maryland, USA, April 28 & 29, 2014, http://ontolog.cim3.net/OntologySummit/

[B58] Belleau F Nolin M-A Tourigny N Rigault P Morissette J Bio2rdf: towards a mashup to build bioinformatics knowledge systems J Biomed Inform 2008 41 5 706 716 10.1016/j.jbi.2008.03.004 18472304

[B59] Jupp S Stevens R Hoehndorf R Logical gene ontology annotations (goal): exploring gene ontology annotations with owl J Biomed Seman 2012 3 Suppl 1 3 doi:10.1186/2041-1480-3-S1-S3 10.1186/2041-1480-3-S1-S3 PMC3337258 22541594

[B60] Wimalaratne SM Grenon P Hoehndorf R Gkoutos GV de Bono B An infrastructure for ontology-based information systems in biomedicine: Ricordo case study Bioinformatics 2012 28 3 448 450 10.1093/bioinformatics/btr662 22130590

[B61] Tudose I Hastings J Muthukrishnan V Owen G Turner S Dekker A Kale N Ennis M Steinbeck C Ontoquery: easy-to-use web-based owl querying Bioinformatics 2013 29 22 2955 2957 doi:10.1093/bioinformatics/btt514. http://bioinformatics.oxfordjournals.org/content/29/22/2955.full.pdf+html 10.1093/bioinformatics/btt514 24008420 PMC3810857

[B62] Cote R Jones P Apweiler R Hermjakob H The ontology lookup service, a lightweight cross-platform tool for controlled vocabulary queries BMC Bioinformatics 2006 7 1 97 doi:10.1186/1471-2105-7-97 10.1186/1471-2105-7-97 16507094 PMC1420335

[B63] Noy NF Shah NH Whetzel PL Dai B Dorf M Griffith N Jonquet C Rubin DL Storey M-AA Chute CG Musen MA Bioportal: ontologies and integrated data resources at the click of a mouse Nucleic Acids Res 2009 37 Web Server issue 170 173 doi:10.1093/nar/gkp440 10.1093/nar/gkp440 PMC2703982 19483092

[B64] Xiang Z Mungall C Ruttenberg A Hea Y Bodenreider O, Martone ME, Ruttenberg A Ontobee: a linked data server and browser for ontology terms Proceedings of International Conference on Biomedical Ontology, ICBO 2011. Buffalo, NY, USA, July 26–30, 2011; 2011 279 281

[B65] Hunter L Lu Z Firby J Baumgartner WA Johnson HL Ogren PV Cohen KB OpenDMAP: an open source, ontology-driven concept analysis engine, with applications to capturing knowledge regarding protein transport, protein interactions and cell-type-specific gene expression BMC Bioinformatics 2008 9 78 10.1186/1471-2105-9-78 18237434 PMC2275248

[B66] Rebholz-Schuhmann D Grabmuller C Kavaliauskas S Harrow I Kapushevsky M Westaway M Woollard P Wilkinson N Strutt P Braxtenthaler M Hoole D Wilson J o‘Beirne R Clark D Kidd RR Semantic integration of gene-disease associations for diabetes type 2 from literature and biomedical data resources Drug Discovery Today 2013 http://www.sciencedirect.com/science/article/pii/S1359644613003917 10.1016/j.drudis.2013.10.024 24201223

[B67] Ferreira JD Hastings J Couto FM Exploiting disjointness axioms to improve semantic similarity measures Bioinformatics 2013 29 21 2781 2787 doi:10.1093/bioinformatics/btt491. http://bioinformatics.oxfordjournals.org/content/29/21/2781.full.pdf+html 10.1093/bioinformatics/btt491 24002110

